# Case Report: Rare meets rare—miliary prostate cancer brain metastasis

**DOI:** 10.3389/fonc.2025.1649587

**Published:** 2025-11-18

**Authors:** Jaspreet K. Gill, Jay Detsky, Eyal Golan, Robert Yeung, Audrey Shiner, Urban Emmenegger

**Affiliations:** 1 Division of Medical Oncology, Odette Cancer Center, Sunnybrook Health Sciences Centre, Toronto, ON, Canada; 2 Department of Radiation Oncology, Odette Cancer Center, Sunnybrook Health Sciences Centre, Toronto, ON, Canada; 3 Division of Critical Care, Department of Medicine, Mackenzie Health, Toronto, ON, Canada; 4 Department of Medical Imaging, Sunnybrook Health Sciences Center, Toronto, ON, Canada; 5 Institute of Medical Science, University of Toronto, Toronto, ON, Canada; 6 Temerty Faculty of Medicine, University of Toronto, Toronto, ON, Canada

**Keywords:** miliary, brain, metastasis, prostate, cancer

## Abstract

**Introduction:**

Miliary brain metastasis (MM), consisting of innumerable miliary lesions in perivascular location, is a rare disease entity with an estimated incidence of 3.8% among patients with brain metastasis (BM). Similarly, with an approximated incidence of less than 2%, prostate cancer (PC)-related BM is also an infrequent presentation; however, it is more common in patients with neuroendocrine differentiation. To the best of our knowledge, only one other case of MM secondary to PC has been reported. This case report discusses two additional cases of PC-related MM, a condition otherwise predominantly observed secondary to pulmonary adenocarcinoma.

**Case presentations:**

The first case describes a patient in his 60s known for metastatic PC with suspected neuroendocrine differentiation and presenting with musculoskeletal pain, lethargy, and status epilepticus. Contrast-enhanced computer tomography (CT) angiogram and magnetic resonance imaging (MRI) of the head and neck revealed diffuse and innumerable foci in the cerebral hemispheres, brainstem, and cerebellum. The second case discusses a similarly aged male patient with biopsy-proven *de novo* mixed adenocarcinoma/small cell neuroendocrine PC and with symptoms consisting of significant weakness, aphasia, confusion, and decreased level of consciousness. Non-contrast-enhanced CT imaging of the brain did not reveal MM; however, a follow-up contrast-enhanced MRI detailed miliary lesions in the cortex, white matter, deep gray nuclei, brainstem, and cerebellum. Both patients expired within a couple of weeks from admission.

**Conclusion:**

Given its rarity, notably in patients with PC, there are no specific and established diagnostic criteria for MM, a condition with ominous prognosis seemingly related to neuroendocrine differentiation in men with PC.

## 1 Introduction

Miliary brain metastasis (MM), also termed “carcinomatous encephalitis”, is a type of cerebral metastasis characterized by small and innumerable foci predominantly observed in a perivascular distribution in the gray and white matter (1, 2). Patients with MM typically present with seizures, hemiparesis, and cognitive impairment (1–3). In the first report of MM published in 1951, Madow and Alpers approximated an incidence of 3.8% of MM among patients with brain metastases, thereby describing the condition as very rare (1).

MM is most frequently observed in patients with pulmonary adenocarcinoma (2–5). This predilection may, in part, reflect the association between miliary metastases and epidermal growth factor receptor (EGFR)-mutant non-small cell lung cancers, as demonstrated in a population-based study (6). However, beyond lung malignancies, isolated case reports have also implicated neuroendocrine differentiation of primary tumors in driving MM—for instance, a case of gastric small cell carcinoma presented with diffuse MM (7). To the best of our knowledge, there is only one reported case describing MM secondary to prostate cancer (PC) in a patient with neuroendocrine prostate cancer (NEPC) (8). While PC brain metastasis is uncommon, with an estimated incidence of less than 2% (9), the rate is higher in patients with NEPC (10). The present report describes two cases of PC-related MM secondary to postulated (case 1) and biopsy-proven (case 2) NEPC, respectively.

## 2 Case presentations

### 2.1 Case 1—musculoskeletal pain, lethargy, and seizures in a patient with metastatic prostate cancer

A patient in his mid-60s with a 2-week history of increased musculoskeletal pain and lethargy, eventually presenting with status epilepticus, was admitted to an external hospital. In the prior 12 years, he was diagnosed with grade group 1 (Gleason Score 3 + 3) T1c PC with an elevated prostate-specific antigen (PSA) level of 8.53 ng/mL and was treated with low-dose-rate brachytherapy. The patient reported that he was an ex-smoker, consumed low doses of alcohol, and had a history of treated hypertension and hyperlipidemia. At 8 years after the initial therapy, he developed local recurrence, and a biopsy revealed grade group 4 (Gleason Score 4 + 4) PC before he underwent salvage focal high-dose-rate brachytherapy. Nearly a year after salvage brachytherapy, he was started on intermittent androgen deprivation therapy (ADT) for 9 months, three injections of goserelin at 10.8 mg sc q12weeks, for biochemical progression (PSA, 15.7 ng/mL). At 10 months after pausing, ADT was restarted because of a PSA elevation to 23.62 ng/mL amid a testosterone level of 12.6 nmol/L and a T12 lesion with Bilsky 1a epidural disease as well as a small osseous metastasis at S1. The two skeletal lesions were treated with stereotactic body radiation therapy, and apalutamide was added, resulting in a PSA nadir of 0.16 ng/mL half a year later.

Routine contrast-enhanced CT images of the chest and abdomen obtained a year after the start of apalutamide revealed new pulmonary nodules, multiple destructive osseous lesions, including a 28-mm deposit in the coracoid process of the scapula on the left with a minimally displaced pathological fracture, and several new lucent foci in the thoracic spine. There was also significant locoregional disease recurrence with a large lesion contiguous with the prostate involving the left seminal vesicle and left posterior bladder wall, metastatic left pelvic lymph nodes, and new liver metastases. Given the presence of extensive metastasis, including to the liver and lung, and the rapid appearance of widespread skeletal pain despite a comparatively low PSA of 1.27 ng/mL, neuroendocrine transformation was suspected (11). Thus, it was decided to treat the patient with six cycles of etoposide and carboplatin chemotherapy, resulting in a partial biochemical and radiological response as well as resolution of skeletal pain. However, following a 3-month chemotherapy break, the patient experienced rapid-onset reappearance of skeletal pain combined with lethargy.

**Figure 1:**
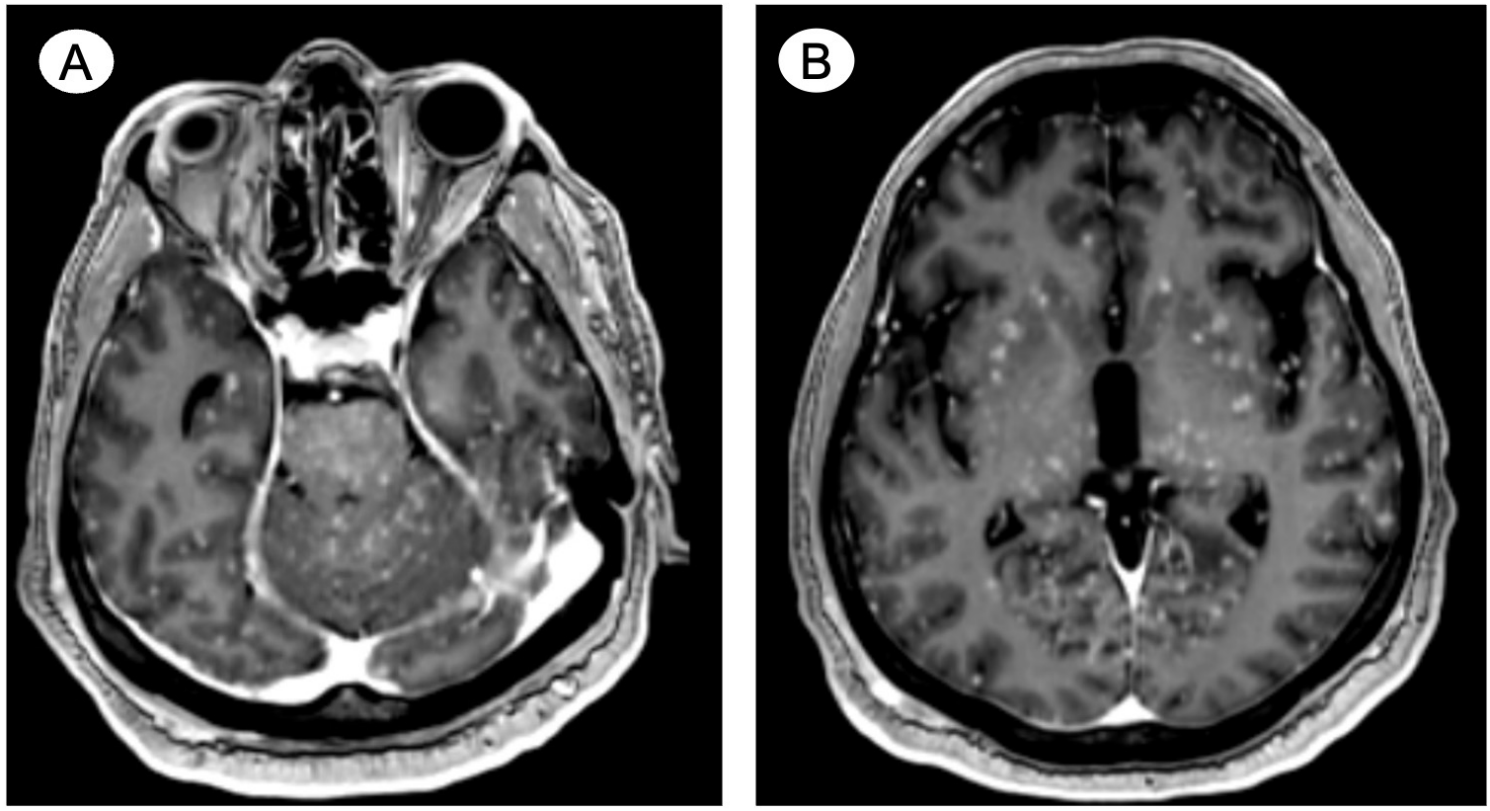
Case 1—Postulated treatment-emergent neuroendocrine prostate cancer with miliary spread to the brain. Contrast-enhanced axial MRI reveals small, diffuse, and innumerable foci predominantly located in the cortical gray matter in **(A)** the brainstem and cerebellum and **(B)** throughout the cerebral hemispheres bilaterally.

Within less than 3 weeks of symptom reappearance, the patient presented to a local emergency room with generalized tonic–clonic seizures. Due to treatment-refractory seizure activity, he was intubated and admitted to the intensive care unit. An intravenous contrast-enhanced CT angiogram and MRI scan of the head and neck detailed small, diffuse, and innumerable foci predominantly located in the cortical gray matter in the cerebral hemispheres bilaterally, with several foci also located in the brainstem and cerebellum (shown in **Figure 1**). The CT also detailed new osseous metastases within the upper thorax. Based on the imaging findings, the patient was diagnosed with MM. While a more detailed diagnostic workup and radiation therapy were considered, due to a lack of clinical improvement and accounting for a presumed guarded prognosis, he was eventually extubated and comfort care was initiated. The patient died nearly two weeks after MM diagnosis.

### 2.2 Case 2—decreased level of consciousness and weakness in a 69-year-old male patient with mixed adenocarcinoma/small cell neuroendocrine prostate cancer

A patient in his late 60s with a 5-day history of significant weakness, aphasia, confusion, and decreased level of consciousness was admitted to the hospital. The patient had exhibited elevated PSA levels for a decade but declined a prostate biopsy until less than a year prior to his admission, when he was diagnosed with mixed adenocarcinoma/small cell neuroendocrine PC. The pathology report detailed 25% adenocarcinoma and 75% NEPC components and a Ki-67 index greater than 95%. Molecular analysis documented the absence of pathogenic variants of BRCA1, BRAC2, ATM, and PALB2. At the time of initial diagnosis, there was metastatic spread to the liver, lungs, pelvic lymph nodes, and bones. The patient reported that he was a non-smoker, did not consume alcohol for the past 8 years, took medication for arterial hypertension, dyslipidemia, and gastroesophageal reflux, and had suffered a posttraumatic subdural hematoma over 30 years ago with complete clinical recovery. Chemotherapy consisting of four cycles of carboplatin/etoposide resulted in a partial response of lung metastases and stability of bone metastases; however, liver and nodal metastases progressed. Subsequently, four cycles of carboplatin/paclitaxel therapy were administered, achieving a partial radiological response.

**Figure 2:**
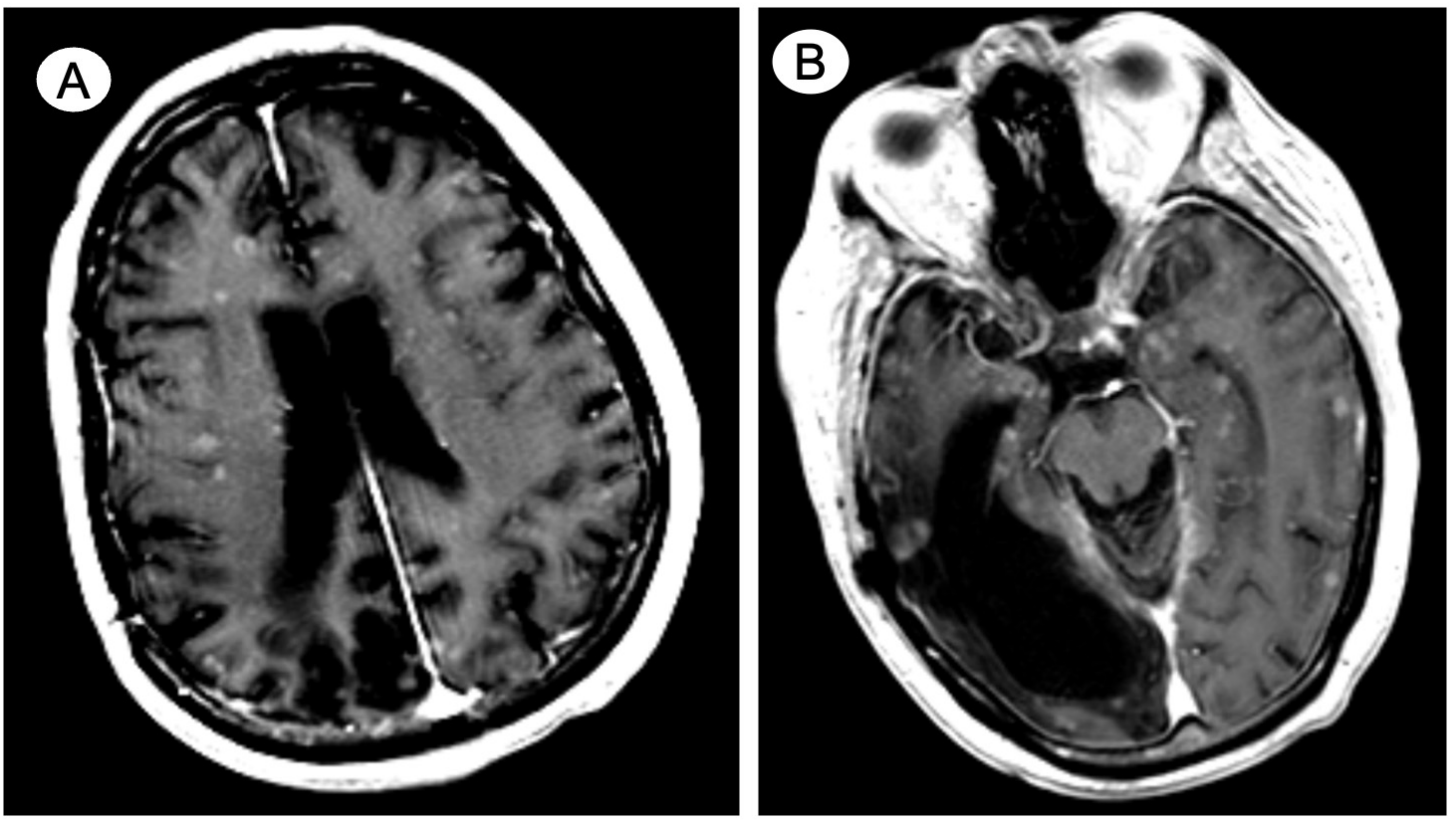
Case 2—*De novo* widely metastatic mixed adenocarcinoma/small cell neuroendocrine prostate cancer with miliary spread to the brain. Contrast-enhanced axial MRI details innumerable miliary foci diffusely spread throughout the cortex and white matter, involving **(A)** both cerebral hemispheres and **(B)** the deep gray nuclei and brainstem as well as cerebellum (not shown). Encephalomalacia in the right temporal and parietal lobes is noted **(B)** due to remote posttraumatic subdural hematoma.

During the fourth cycle of carboplatin/paclitaxel, the patient was admitted for septic shock secondary to *Klebsiella pneumoniae* bacteremia complicated by radiologically suspected spondylodiscitis (T8-T9). Shortly following 6 weeks of intravenous antibiotic therapy, he developed confusion, aphasia, and a decreased level of consciousness leading to hospital admission. This prompted for non-contrast-enhanced CT imaging of the brain that did not reveal any acute intracranial findings. However, a follow-up contrast-enhanced MRI on the next day disclosed innumerable miliary foci diffusely spread throughout the cortex and white matter, deep gray nuclei, brainstem, and cerebellum (shown in **Figure 2**). Consistent with the patient’s past medical history, the MRI also reported encephalomalacia in the right temporal and parietal lobes. The workup for infectious causes, including blood and mycobacterial cultures, remained negative. CT imaging of the chest was not suggestive of acute tuberculosis infection. Moreover, lumbar punctures were unsuccessful, complicated by the persistent confusion of the patient. His overall health declined rapidly, and the family agreed to initiate comfort measures. At 2 weeks after admission, the patient expired.

## 3 Discussion

**Figure 3:**
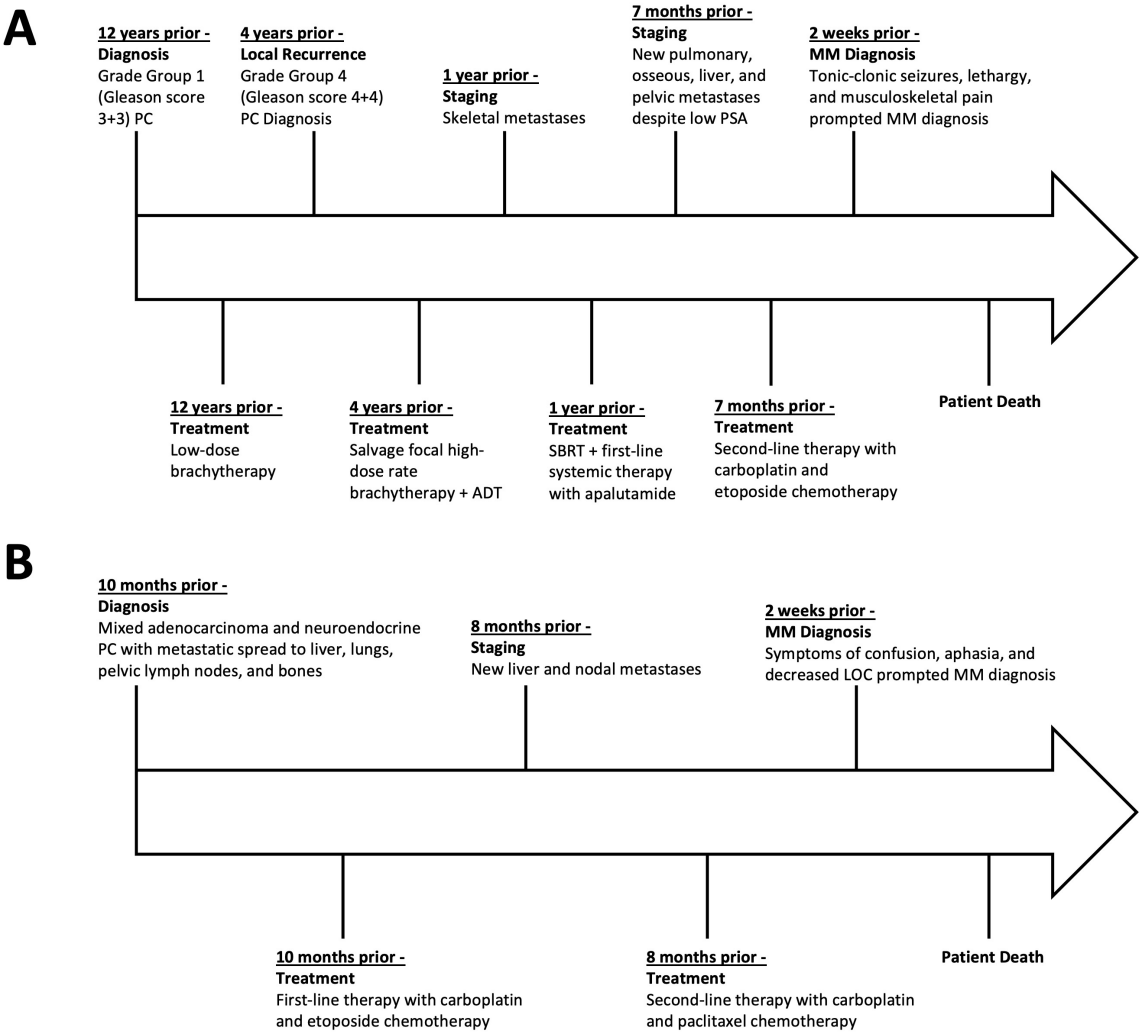
Timeline of disease course, treatments, and key events in case 1 **(A)** and case 2 **(B)**, presented relative to the time prior to the patients’ death. PC, prostate cancer; ADT, androgen-deprivation therapy; SBRT, stereotactic-body radiation therapy; PSA, prostate-specific antigen; MM, miliary brain metastasis.

This report describes two cases of MM secondary to postulated treatment-emergent NEPC (case 1) and biopsy-proven *de novo* mixed adenocarcinoma/small cell NEPC (case 2) (11, 12). The graphic timeline of events associated with the two cases are displayed in **Figure 3**. MM is a rare form of cerebral metastasis presenting with diffuse spread of small and innumerable punctate tumor nodules typically observed in the perivascular distribution of the gray and white matter (1, 2). As per published reports, survival after MM diagnosis ranges from weeks to months; hence, the prognosis for the condition is poor irrespective of the treatment administered (3, 4).

Contrast-enhanced MRI or CT as well as histopathological examination are the most commonly used diagnostic tools, but there are no validated radiological diagnostic criteria for MM diagnosis (2, 4). In a case series analysis identifying MM in breast cancer patients, Bashour et al. proposed imaging criteria consisting of two key requisites for MM diagnosis: first, the presence of at least 20 observable lesions on at least two non-contiguous MRI slices or at least 10 lesions on at least two non-contiguous CT slices; and secondly, the distribution of lesions bilaterally and in both the supratentorial and infratentorial regions (4). In case 1, a contrast-enhanced CT angiogram and MRI scan were diagnostic by revealing extensive miliary spread. Conversely, in case 2, the initial non-contrast-enhanced CT performed in the emergency department was unsuccessful in disclosing the miliary spread, whereas the MRI scan performed a day later revealed MM as per the Bashour criteria. Histopathological examination (e.g., biopsy or autopsy) is necessary to diagnose MM with absolute certainty; however, tissue sampling is often not feasible or deemed inappropriate given the usually highly symptomatic clinical presentation of patients with MM (2). Importantly, differential diagnoses, notably infectious etiologies (e.g., tuberculosis), must be considered (2).

In both cases described herein, differential diagnoses—including infectious and inflammatory etiologies, were carefully considered. In case 1, while extensive infectious workup was not sought, the rapid clinical decline with treatment-refractory seizures favored a metastatic etiology. In case 2, blood and mycobacterial cultures were negative, chest CT was unremarkable for acute tuberculosis infection, and lumbar puncture attempts were unsuccessful; nonetheless, the absence of systemic infection markers, together with the imaging findings, made infectious encephalitis or tuberculosis unlikely. Applying the Bashour criteria, both cases satisfied the MRI- and CT-based criteria accompanied by widespread innumerable lesions in both supratentorial and infratentorial regions. Although histopathological confirmation was not feasible, the clinical and radiological profiles of both cases strongly supported MM. Importantly, a recent systematic review by Garg et al. emphasized that while tuberculosis remains the most common cause of miliary brain lesions, malignancy is the second most frequent etiology, typically arising at advanced stages of the primary disease (13). Patients with malignant miliary lesions are generally older, typically present with encephalopathy rather than fever or meningitic features, and face a markedly poorer prognosis compared to infectious causes (13). Both of our patients conformed to this pattern with rapid neurological decline, absence of systemic infectious signs, and death within weeks of diagnosis.

MM most commonly presents itself secondary to lung malignancies, which have a high propensity for brain metastases, in general (2–5, 14). In 2020, a case report and literature review by Santos Vázquez et al. identified a total of 26 reported cases of miliary CNS metastases, and 16 of the 26 cases (61.5%) identified were secondary to lung cancer, with 15 of the 16 primary tumors being adenocarcinomas and the remaining malignancy being a small cell carcinoma (5). As per published evidence, patients with MM commonly present with seizures, hemiparesis, and cognitive impairment (1–3). The patient described in case 1 most notably experienced status epilepticus, increased musculoskeletal pain, and lethargy, whereas the patient in case 2 presented with significant weakness, decreased level of consciousness, aphasia, and confusion.

Brain metastasis secondary to prostate cancer is uncommon, with a reported incidence of 2% or less (9). With MM being a rare type of brain metastasis, to the best of our knowledge, only one additional case of MM secondary to prostate cancer has been reported to date (8). In this case report by Melendez-Zaidi et al., a 70-year-old male patient presented with treatment-emergent NEPC with metastasis to bones, liver, and lungs as well as fatigue, impaired memory, and altered behavior (8). An MRI detailed innumerable foci in the supratentorial and infratentorial regions, and an autopsy revealed countless metastatic lesions in the perivascular regions staining positive for PSA, NKX3.1, and synaptophysin (8). With respect to case 1 described herein, neither *in vivo* nor *post-mortem* histopathological diagnosis was pursued, the former owing to the severity of his clinical presentation. In case 2, a lumbar puncture was attempted but was not successful. To further compare the three cases, all three patients presented with either clinically suspected or clinicopathologically diagnosed NEPC. The latter may arise *de novo* (1% of PC patients) or present as a treatment-emergent disease after androgen-receptor-targeted therapy, estimated to occur in 15%–20% of advanced PC cases (15, 16). The mechanisms of neuroendocrine transformation involve loss of TP53 and RB1, lineage plasticity, and activation of stem-cell-like reprogramming pathways, resulting in an aggressive clinical phenotype with propensity for widespread visceral metastases and comparatively low PSA levels despite high disease burden (15, 16). Notably, since NEPC is associated with higher rates of brain metastasis than prostate adenocarcinoma, this may be a plausible explanation for why all three cases of MM secondary to prostate cancer were observed in patients with NEPC (10). However, the molecular underpinning of MM is unknown. The case by Melendez-Zaidi et al. was described as “retinoblastoma protein and microsatellite instable positive” (8). The mixed adenocarcinoma/NEPC of case 2 did not display DNA repair defects involving BRCA1, BRCA2, ATM, or PALB2. While the lack of histopathological documentation of MM is a limitation in both cases reported herein, the scope of future research should not only elaborate on the molecular characterization of MM but also on establishing specific and universally accepted diagnostic criteria of MM. Specifically, in high-risk subgroups such as patients with NEPC, prospective CNS imaging protocols may help identify MM earlier. Additionally, molecular profiling of tissue (when feasible) could provide insights into the biology of MM and clarify why NEPC appears predisposed to this pattern of spread.

## 4 Conclusion

Brain metastasis secondary to PC is rare (9). With MM being a very uncommon presentation of cerebral metastasis (1), to the best of our knowledge, this report details the second and third reported cases of MM secondary to PC, notably all suspected to be driven by neuroendocrine differentiation and associated with poor prognosis.

## Data Availability

The datasets presented in this article are not readily available due to confidentiality reasons. All relevant data generated during the preparation of this manuscript are included in this article. Further inquiries can be directed to the corresponding author. Requests to access the datasets should be directed to urban.emmenegger@sunnybrook.ca.
